# Effects of lovastatin on breast cancer cells: a proteo-metabonomic study

**DOI:** 10.1186/bcr2485

**Published:** 2010-03-05

**Authors:** Jelena Klawitter, Touraj Shokati, Vanessa Moll, Uwe Christians, Jost Klawitter

**Affiliations:** 1 Department of Anesthesiology, Clinical Research and Development, University of Colorado Denver, 12401 East 17th Avenue, Aurora, CO, 80045, USA; 2 Eurofins Medinet Inc., 1999 North Fitzsimons Parkway, Aurora, CO, 80045, USA

## Abstract

**Introduction:**

Statins are cholesterol-lowering drugs with pleiotropic activities including inhibition of isoprenylation and reduction of signals driving cell proliferation and survival responses.

**Methods:**

In this study we evaluated the effects of lovastatin acid and lactone on breast cancer MDAMB231 and MDAMB468 cells using a combination of proteomic and metabonomic profiling techniques.

**Results:**

Lovastatin inhibited proliferation of breast cancer cell lines. MDAMB231 cells were more sensitive to its effects, and in most cases lovastatin acid showed more potency towards the manipulation of protein expression than lovastatin lactone. Increased expression of Rho inhibitor GDI-2 stabilized the non-active Ras homolog gene family member A (RhoA) leading to a decreased expression of its active, membrane-bound form. Its downstream targets cofilin, CDC42 and G3BP1 are members of the GTPase family affected by lovastatin. Our data indicated that lovastatin modulated the E2F1-pathway through the regulation of expression of prohibitin and retinoblastoma (Rb). This subsequently leads to changes of E2F-downstream targets minichromosome maintenance protein 7 (MCM7) and MutS homolog 2 (MSH2). Lovastatin also regulated the AKT-signaling pathway. Increased phosphatase and tensin homolog (PTEN) and decreased DJ-1 expression lead to a down-regulation of the active pAkt. Lovastatin's involvement in the AKT-signaling pathway was confirmed by an upregulation of its downstream target, tumor progressor NDRG1. Metabolic consequences to lovastatin exposure included suppression of glycolytic and Krebs cycle activity, and lipid biosynthesis.

**Conclusions:**

The combination of proteomics and metabonomics enabled us to identify several key targets essential to the antitumor activity of lovastatin. Our results imply that lovastatin has the potential to reduce the growth of breast cancer cells.

## Introduction

Breast cancer is the second leading cause of cancer death in women. There are currently no effective therapies for advanced breast cancer, with treatment primarily aimed at palliation of symptoms and improvement of overall survival. Healthy women at high risk of breast cancer are the focus of prevention, whereas current chemotherapy targets women after a positive diagnosis. Prevention in at risk, but healthy, women requires efficacious drugs with a good long-term safety and tolerability profile. Statins fit these criteria [1-6].

Statins are competitive inhibitors of 3-hydroxy-3-methylglutaryl coenzyme reductase (HMG-CoA). They reduce cholesterol synthesis by blocking the conversion of HMG-CoA to mevalonate [7]. The end products of the mevalonate pathway are required for a number of essential cellular functions. The end products include: sterols, involved in membrane integrity and steroid production; ubiquinone (coenzyme Q), involved in electron transport and cell respiration; farnesyl and geranylgeranyl isoprenoids, involved in covalent binding of proteins to membranes; dolichol, which is required for glycoprotein synthesis; and isopentenyladenine, essential for certain tRNA functions and protein synthesis [8,9].

HMG-CoA reductase inhibitors have been shown to inhibit cellular proliferation and induce apoptosis and necrosis in several experimental settings including that of breast cancer, thus making them potential anticancer agents [10-12]. Induction and enhancement of reactive oxygen species (ROS) formation has been explored as a possible cause of cytotoxicity of statins in breast cancer cells [13]. Stimulation of nitric oxide synthase (iNOS) and the subsequent increase in nitric oxide (NO) levels may also play a role in the pro-apoptotic and anti-proliferative effects of statins on breast cancer cells [14]. Several cell signaling pathways seem to be involved in the inhibition of cell proliferation and statin-induced cancer cell death, including FAK/ERK pathways [15], increased expression of p21, p27 and activated caspase-3, and changes in the expression of several cyclin-dependent kinases [16].

Recent clinical data show that statins may influence the phenotype of breast tumors, suggesting a new potential strategy for breast cancer prevention, that of combining statins with agents that prevent estrogen receptor (ER)-positive cancer (tamoxifen, aromatase inhibitors) [1]. Another study suggested statin treatment following breast cancer diagnosis decreases the risk of recurrence, and a further decline in correlation to the duration of statin use [2]. Lovastatin is orally administered to patients in its lactone form. However, after absorption, lovastatin is quickly converted into its open acid form and, as with most statins, lovastatin is present in plasma as the active acid that is responsible for HMG-CoA inhibition and two orders of magnitude more lipophilic lactone. As both forms have distinct physicochemical properties and potentially different mechanisms of action, both are studied here.

In order to gain more insight into the anticancer activity and mechanism of action of statins in breast cancer cells, our study employed a combination of proteomics-based and nuclear magnetic resonance (NMR)-based metabonomics techniques. We identified new key targets of lovastatin, and revealed involvement of several regulatory cellular pathways in the cytotoxic effects of lovastatin on breast cancer cell lines.

## Materials and methods

### Reagents

Tris-HCl, sodium chloride, EDTA, NP-40, sodium deoxycholate, urea, thiourea, SDS, 20% glycerol, methanol, acetic acid and iodacetamide were purchased from Sigma-Aldrich (Allentown, PA, USA). Protease and phosphate inhibitors were from Pierce Biotechnology (Rockford, IL, USA); immobilized pH gradient (IPG) buffer pH 3 to 11 was from GE Healthcare (Piscataway, NJ, USA) and dithiothreitol (DTT) was from USB (Cleveland, OH, USA). Lovastatin (in its lactone and hydroxy acid form) was purchased from Toronto Research Chemicals (North York, Ontario, Canada). 3-(4,5-dimethylthiazol-2-Yl)-2,5-diphenyltetrazolium bromide (MTT) cell growth assay kits were from Millipore (Billerica, MA, USA).

### Cell culture and treatments

MDAMB468 and MDAMB231 cell lines were from American Type Culture Collection (ATCC) and propagated according to the instructions provided. Both cell lines are ER negative, and for this study relevant differences were that MDAMB468 cells lack expression of retinoblastoma (Rb), phosphatase and tensin homolog (PTEN) and steroid and xenobiotic receptor (SXR) proteins.

For proteomics studies, cells were treated for 48 hours with 8 μg/mL lovastatin lactone or hydroxy acid. MTT assays were performed prior to proteomics studies for IC50 determination. To investigate the effects of isoprenoid intermediates of the cholesterol biosynthetic pathway, in particular geranylgeranyl diphosphate (GGPP), farnesyl pyrophosphate (FPP) and mevalonic acid on the proliferation of cells treated with lovastatin, MDAMB231 and MDAMB468 cells were treated with 2 μg/mL, 4 μg/mL and 8 μg/mL lovastatin acid or lactone and were 'rescued' by addition of 10 μM GGPP, 100 μM mevalonic acid or 10 μM FPP.

### MTT assay

The cells were cultured in 96-well plates. Treatment occurred with lovastatin in its lactone or acid form or with a combination of lovastatin with GGPP, FPP or mevalonic acid for 48 hours. During the last four hours, 0.02% MTT solution was added and the reaction was stopped with isopropanol and 5% acetic acid. The production of purple formazan in cells treated with an agent was measured relative to the production in control cells and dose-response curves were generated with a Perkin Elmer (Waltham, MA, USA) ELISA plate reader at 525 nm.

IC_50 _values were estimated using the Prism software (GraphPad Software Inc., Version 4.0, San Diego, CA, USA).

### Two-dimensional gel electrophoresis

For proteomics studies, cells were washed twice with ice-cold PBS followed by a collection in modified RIPA lysis buffer (50 mM Tris-HCl, pH 7.4; 150 mM NaCl; 1 mM EDTA; 1% NP-40 (v/v); 0.25% sodium deoxycholate (v/v); protease and phosphatase inhibitor cocktail). After complete solubilization, cell extracts were subjected to purification using a 2-D clean-up kit (GE Healthcare, Piscataway, NJ, USA) in accordance with the manufacturer's instructions. The final solubilization was performed in chaotropic lysis buffer containing 7 M urea, 1 M thiourea, 50 mM DTT, 0.4% IPG buffer pH 4 to 7, protease and phosphatase inhibitors. The protein concentrations were determined using a BioRad Bradford protein assay kit (BioRad, Hercules, CA, USA). Samples of 300 μg cell extract (in 200 μL) were loaded onto Immobiline DryStrips (11 cm, pH 3 to 8, GE Healthcare, Piscataway, NJ, USA). Isoelectric focusing was performed on a Protean IEF cell (Biorad, Hercules, CA, USA) with the following voltage program: rehydration: 50 V, 12 hours; 1000 V, 2 hours (gradient); 6000 V, 4 hours (gradient); 8000 V, 6 hours (rapid), maximal current 50 uA per strip. Strips were equilibrated in 20 mL rehydration buffer (6 M urea, 50 mM Tris-HCl pH 8.8, 2% SDS, 20% v/v glycerol) containing 10 mg/mL DTT and 25 mg/mL iodacetamide for 20 minutes each. The second dimension was performed using a Mini-Protean Dodeca chamber (Biorad, Hercules, CA, USA) on 10.5 to 14% Criterion Tris-HCl gels (IPG + 1 well, 11 cm, Biorad, Hercules, CA, USA). Gels were washed with nanopure water and with Biosafe Coomassie-Blue Stain (Biorad, Hercules, CA, USA) or fixed for one hour in 50% methanol, 10% acetic acid and stained with Sypro Ruby protein gels stain (Invitrogen, Carlsbad, CA, USA) overnight. Prior to imaging Coomassie-Blue stained gels were washed in nanopure water for up to 24 hours and imaged on LabScan Image Scanner (GE Healthcare, Piscataway, NJ, USA) with 900 dpi. Sypro Ruby-stained gels were washed twice in 10% methanol and 7% acetic acid for one hour each and imaged on a Typhoon 8600 imager (Amersham Pharmacia Biotech, Piscataway, NJ, USA) with 532 nm laser wavelength.

Gel image analysis was carried out using the ImageMaster 2D Platinum II software version 5.0 (GE Healthcare, Piscataway, NJ, USA). The spot auto-detect function was used for all group comparisons using identical parameters. Groups were matched automatically and corrected manually if necessary. Differences in protein expression were identified using the relative volume (%Vol) option of the software. This option allows the data to be independent of experimental variations between gels caused by differences in loading or staining. Relative volume was calculated as follows [17,18]:

 with Vol_s _being the volume of spot *s *in a gel containing *n *spots.

Raw spot values were normalized using the software's ratio option according to the following equation [17-19]:

 with central tendency being the mean of spot *s*.

Changes in average volume larger than ± 40% of the average spot volume and the significance level of *P *< 0.05 (control vs. treated group) was the criterion used for excision. Four replicates were used for each control, lovastatin lactone or acid treatment, respectively.

### In-gel digestion

Proteins from excited gels spots were digested using a modification of the method by Havlis [20]. Briefly, spots were destained with acetonitrile and 100 mM ammonium bicarbonate (50/50 v/v), contracted with 100% acetonitrile and then vacuum dried. Spots were rehydrated with 50 μg/ml trypsin (sequencing grade II, Worthington, Lakewood, NJ, USA) and incubated for 10 minutes on ice. Excess liquid was removed and 50 mM ammonium bicarbonate added prior to overnight incubation at 37°C. The supernatants were collected and pooled with two additional extracts using 1% formic acid with 30% acetonitrile. Pooled extracts were vacuum concentrated to approximately 10 μL and stored at -80°C until mass spectrometry analysis.

### LC-MS/MS analysis of tryptic digests

The analysis of tryptic digests was performed using a 4000 QTRAP liquid chromatography (LC) mass spectrometry (MS)/MS system (Applied Biosystems, Foster City, CA, USA) equipped with a Dionex Ultimate 3000 nano-LC system (Dionex Corporation, Sunnyvale, CA, USA).

Peptides were loaded onto an enrichment column (C18PM, LC packings 0.3 mm ID) with 3% acetonitrile (ACN) and 0.05% trifluoroacetic acid (TFA) at a flow rate of 4 μL/min. After activation of a switching valve, the peptide mixture was back-flushed from the enrichment onto the analytical column (Zorbax 300SB C18, 3.5 um, 150 × 75, Agilent Technologies, Palo Alto, CA, USA) using a gradient. Solvent A was 0.1% formic acid and solvent B was 80% CAN and 0.1% formic acid. The flow rate was 400 nL/min. Buffer B was increased from 5% to 8% in one minute and then from 8% to 45% over 39 minutes. Finally, solvent B was increased to and held at 80% for the next five minutes, after which the settings were returned to initial conditions. Spectra were collected over an *m/z *range of 350 to 2200 Da. Three MS/MS spectra were collected for the three most abundant *m/z *values. Then those were excluded from analysis for one minutes and the next three most abundant *m/z *values were selected for fragmentation.

### Protein identification using database searching

Proteins were identified by searching the databases of the NCBInr (National Center for Biotechnology Information, non-redundant) and SwissProt (Swiss Institute of Bioinformatics) using ProteinPilot 2.0 with paragorn algorithm (Applied Biosystems, Foster City, CA, USA) software. Parameters used in the database search were as follows: biological modifications; fixed modification: iodacetamide alkylation of Cys; detected protein threshold: more than 1 (90%); thorough ID.

### Cell extraction for nuclear magnetic resonance spectroscopy

For NMR experiments, the cells were incubated with 5 mmol/L (1-^13^C) glucose (Cambridge Isotope Laboratories, Andover, MA, USA) for the last five hours prior to the perchloric acid (PCA) extraction. All cell extractions were performed using a previously published PCA extraction protocol that allowed for separation of water-soluble and lipid fractions [21]. Lyophilized water-soluble cell extracts were re-dissolved in 0.5 mL of deuterium oxide, centrifuged and the supernatants neutralized to pH 7.2 in order to allow for precise chemical shift assignments. Lipid fractions were re-dissolved in a 1 mL CD_3_OD/CDCl_3 _mixture (1:2).

### NMR spectroscopy

High-resolution ^1^H and ^13^C-NMR experiments were performed using a Varian INOVA NMR 500 MHz spectrometer equipped with a 5 mm HCN PFG probe (Varian, Palo Alto, CA, USA). For ^1^H-NMR analysis of water-soluble extracts we have used fully relaxed spectra with a standard water presaturation pulse program, whereas for analysis of lipids no presaturation pulse was used. Spectra were obtained at 12 ppm spectral width (10 ppm for lipids), 32 K data arrays, and 64 scans with 90-degree pulses applied every 14.8 seconds. The pool size of metabolites was determined based on fully relaxed ^1^H-NMR spectra of extracts using trimethylsilyl propionic-2,2,3,3,-d_4 _acid (TSP) as an external standard and chemical shift reference (0 ppm). The absolute concentrations of each metabolite [metabolite] were determined and normalized according to cell wet weight, as previously described [22-24] and calculated using the following equation:

where integral_met _is integral of respective metabolite signal divided by the number of protons; integral_TSP _is integral of TSP signal divided by the number of protons; [TSP] is TSP nominal concentration; V_S _is sample volume; wet weight is sample weight.

^13^C-NMR spectra with proton decoupling (composite pulse decoupling) were recorded using the C3-lactate peak at 21 ppm as chemical shift reference (spectral width was 150 ppm, 16 K data arrays, with 20 K scans applied every three seconds). For quantification of absolute concentrations of ^13^C metabolites, calculations were made according to [25,26]. The ^13^C-enrichments in C3-lactate were determined by the heteronuclear spin-coupling pattern in ^1^H-NMR spectra as follows:

where the sum (area [^1^H-^12^C] + area [^1^H-^13^C]) is equivalent to the pool size of lactate. The values were corrected for 1.1% natural abundance ^13^C. ^13^C-enrichments in individual carbons of amino acids were derived from ^13^C-NMR spectra using the known ^13^C-enrichment in lactate:

where A_Met _represents ^13^C carbon peak area of the metabolite, A_n.a. _is its natural abundance signal intensity, and 1.1 is the percentage factor of the ^13^C-isotope. The natural abundance of ^13^C, contributing to the total intensity A_n.a. _(Met), was determined using the known ^13^C-enrichment and natural abundance of lactate and correction for the pool size:

A_Lac _represents the carbon peak area of lactate, [Lac] or [Met] are the pool sizes of lactate or metabolite of interest, respectively, and E_Lac _is the percentage ^13^C-enrichment in lactate. The ^13^C signal intensities were corrected for nuclear Overhauser enhancement effects by comparison with the standard mixture of amino acids.

The absolute amount of ^13^C in specified carbon positions is the product of the pool size multiplied by the fractional ^13^C-enrichment.

### Western blot analysis

Western blot analysis was carried out to validate proteomics 'hits'. Aliquots of frozen extracts were loaded onto Biorad 4 to 12% Bis-Tris Criterion gels and proteins separated using a Biorad Criterion cell electrophoresis system (BioRad, Hercules, CA, USA) operating for approximately two hours at 120 V and then transferred (200 mA, 5 hours) from the gel to an Immobilon-P membrane (Millipore Corporation, Billerica, MA, USA). Membranes were incubated overnight at 4°C with the primary antibody following blocking with 5% milk/BSA in PBS-Tween buffer. Antibodies used in this study included: proliferating cell nuclear antigen (PCNA), prohibitin, E2F-1, RhoGDI, Ras homolog gene family member A (RhoA), pRb, cell division cycle 42 (CDC42), PTEN (Cell Signaling Technology, Inc., Danvers, MA, USA); high-mobility group protein B1 (HMGB1), N-myc downstream regulated gene 1 (NDRG1), DJ-1, pAkt (Abcam, Cambridge, MA, USA); MutS homolog 2 (MSH2), phospho-GTPase activating protein binding protein 1 (G3BP1), pG3BP1 (GenScript, Piscataway, NJ, USA); minichromosome maintenance protein 7 (MCM7) (Biolegend, San Diego, CA, USA). After the membranes were washed three times, the secondary antibody (horseradish peroxidase (various hosts), Pierce, Rockford, IL, USA) was applied for three hours at room temperature. Membranes were subsequently treated with Pierce SuperSignal^® ^West Pico Solution (Pierce, Rockford, IL, USA) in accordance with the method described by the manufacturer's protocol. A UVP BioImaging Systems UV detector (BioImaging Systems, Upland, CA, USA) was used to detect the horseradish peroxidase reaction on the membrane. Densitometry data were normalized by the amount of β-actin.

### Statistical analysis

All numerical data is presented as mean ± standard deviation from replicate experiments. Student's t test, or when applicable one-way analysis of variance (ANOVA) were used to determine differences between groups. Tukey's test was used as a *post-hoc *test in combination with ANOVA to test for significances among groups. The significance level was set at p < 0.05 for all tests (SigmaPlot-version 11.0, Systat Software, Point Richmond, CA, USA) and PASW version 18.0 (SPSS Inc., Chicago, IL, USA).

## Results

### Lovastatin inhibits cell proliferation

**Figure 1:**
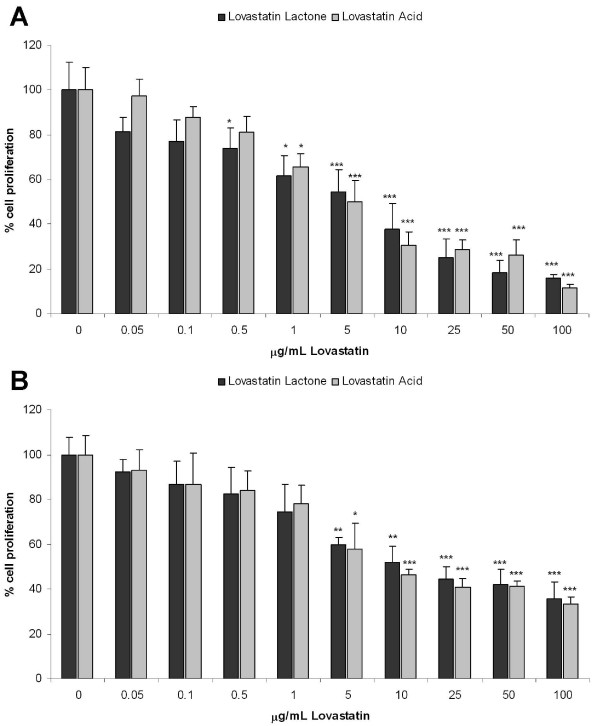
**Cell proliferation of human breast cancer cell lines**. **(a) **MDAMB231 and **(b) **MDAMB468. Cells were treated with increasing concentrations of lovastatin lactone or lovastatin acid (μg/mL) for 48 hours. Data are presented as mean ± standard deviation (n = 5) *P < 0.05;**P < 0.05; ***P < 0.001.

Lovastatin induces inhibition of cell proliferation in MDAMB468 and MDAMB231 cells (Figure 1). The lovastatin hydroxy acid form was slightly more effective in both cell lines with a half maximum inhibition concentration (IC_50_) of 8 μg/mL in MDAMB468 and 5 μg/mL in MDAMB231 cells, whereas the IC_50 _values for lovastatin lactone were 9 μg/mL and 7 μg/mL, respectively. All subsequent experiments were carried out using 8 μg/mL lovastatin in its lactone or acid form.

In rescue experiments, when cells were co-incubated with lovastatin and GGPP, FPP or mevalonate, only mevalonate and GGPP were able to fully rescue cells from the anti-proliferative effect of lovastatin, whereas FPP could only achieve a partial rescue. Upon GGPP and mevalonate co-exposure with 8 μg/mL lovastatin (acid or lactone), cells regained 92 to 98% of the proliferation rate of control cells, whereas only 67% was regained with the co-administration of lovastatin and FPP.

### Two-dimensional gel electrophoresis and MS analysis of lovastatin-induced changes in the protein expression of breast cancer cells

**Figure 2:**
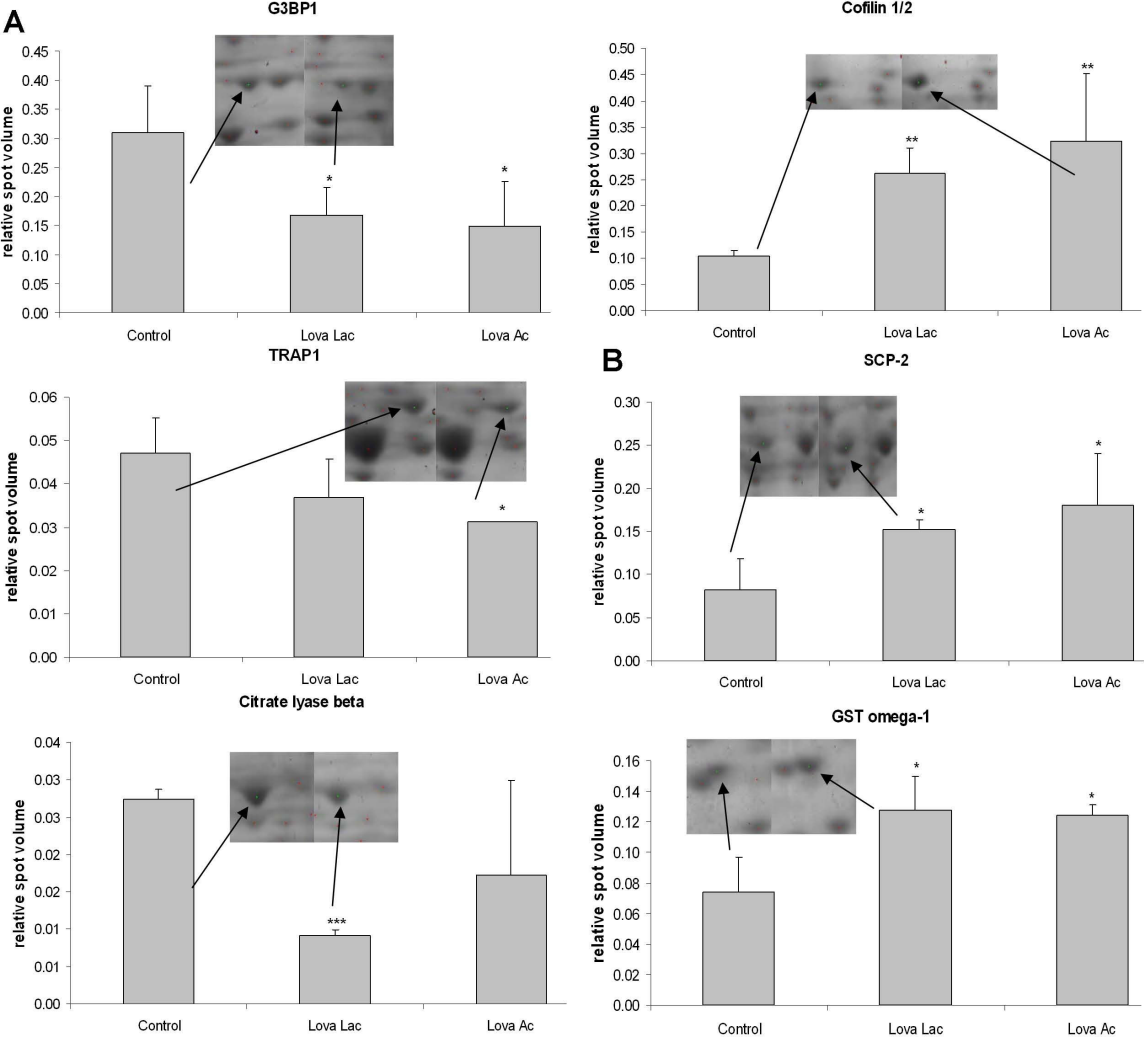
**Changes in expression of proteins involved in (a) regulation of cell cycle and cell death and (b) oxidative and metabolic processes of human MDAMB231 and MDAMB468 cells**. Both cell lines were treated with 8 μg/mL lovastatin lactone (Lova Lac) or lovastatin acid (Lova Ac) for 48 hours. Data represent relative spot volumes (as calculated from two-dimensional gel images of whole cell extracts; data are presented as mean ± standard deviation (n = 5) *P < 0.05;**P < 0.05; ***P < 0.001). Gel spots which showed significant differences in their volume between the control and lovastatin-treated cells were cut-out, proteins were digested and analyzed using liquid chromatography (LC) mass spectrometry (MS)/MS analysis. In MDAMB231 cells they were identified as GTPase-activating protein SH3-domain-binding protein 1 (G3BP1), TNF type 1 receptor-associated protein (TRAP1) and glutathione S-transferase (GST) omega proteins (Table 1a), whereas the spot belonging to citrate lyase beta and sterol carrier protein 2 (SCP-2) originated from MDAMB468 cells (Table 1b). Cofilin1/2 was identified as upregulated in both cell lines. The image and changes as observed in MDAMB231 cells is shown.

In order to obtain a comprehensive view of changes in the protein synthesis in response to lovastatin treatment, proteome analyses using two-dimensional gel electrophoresis were performed on MDAMB468 and MDAMB231 breast cancer cell lines (Figure 2). Both forms of lovastatin (lactone and hydroxy acid form with 8 μg/mL for 48 hours) were used for cell treatment.

### Functional classification of identified proteins

Each identified protein was assigned a functional classification based on the gene ontology annotation in the Database for Annotation, Visualization, and Integrated Discovery (DAVID). The DAVID annotation tool was used for functional clustering and pathway mapping of identified protein hits. A comparison between the expressional changes of spots in the lactone or hydroxy acid group revealed that both chemical forms of lovastatin followed the same directional change through an increase or decrease in the relative protein abundance. For this reason, we combined the treatment groups and these combined protein hits were then subjected to DAVID annotation tool analysis.

Seventy-four proteins were identified as significantly changed upon treatment with 8 μg/mL lovastatin (lactone or acid form) in MDAMB231 cells, and 42 such proteins were identified in MDAM468 cells (Table 1). Despite the stronger response of MDAMB231 cells, impact by lovastatin on the biological processes was similar in both cell lines. For example, the addition of lovastatin not only influenced the major metabolic cellular pathways, such as glycolysis or pentose-phosphate shunt, it also changed expression of proteins involved in the regulation of apoptosis, stress response, cell differentiation and actin-filament morphogenesis. Furthermore, lovastatin lactone and acid exposure-induced changes in cell cycle regulatory proteins and small GTPases mediated signal transduction members.

**Table 1:** Overview of proteins showing significant differences (*P *< 0.05) between control and treated MDAMB468 and MDAMB231 breast cancer cells

*MDAMB468 cells*	Control vs. lactone	Control vs. acid
14-3-3 beta	3.51	1.93
14-3-3 zeta/theta	1.61	1.48
17-beta hydroxysteroid dehydrogenase	0.62	0.47
6-phosphogluconolactonase	1.56	1.5
alpha enolase	1.89	1.37
alpha glucosidase subunit alpha isoform 3	0.64	0.4
ATPase, H+ transporting, lysosomal 70 kDa V1 subunit A	0.30	0.31
ATP citrate lyase beta, mitochondrial	0.33	0.63
Carbonyl reductase [NADPH] 3	3.00	1.56
Chaperonin containing TCP1, subunit 6A	1.34	1.98
chloride intracellular channel 1	1.2	2.12
cofilin 1/2	0.60	0.65
D3-phosphoglycerate dehydrogenase	0.75	0.16
DJ-1	0.54	0.45
EEF1 delta	0.68	0.4
ER-60 protein	2.18	1.56
eukaryotic initiation factor 4A-II	0.35	0.63
eukaryotic translation initiation factor 3 subunit H	0.71	0.49
eukaryotic translation initiation factor 6	4.76	1.57
Ezrin	2.10	4.00
gelsolin precursor	2.73	1.54
glutamate receptor GRIA3	0.24	0.63
glycyl-tRNA synthetase	0.40	0.33
heat shock cognate 71 kDa protein	0.20	0.36
IMMT (mitochondrial inner membrane protein)	3.44	2.32
lamin A/C, isoform CRA_c	0.41	0.47
MAPRE1 protein	0.86	0.4
multidrug resistance-associated protein MGr1-Ag	0.52	0.74
NADH dehydrogenase Fe-S protein 1, 75 kDa	0.56	0.35
nucleoside phosphorylase	1.95	1.31
protein disulfide isomerase associated 6	0.85	0.41
protein disulfide isomerase ER-60	2.13	1.25
RAB8b, member RAS oncogene family	3.16	1.48
RAVER-1 protein	2.17	1.60
splicing factor, arginine/serine-rich 1	0.24	0.71
sterol carrier protein X/2	0.45	0.52
stress-70 protein, mitochondrial precursor (observed pI 5.9; theoretical pI 5.9)	0.65	0.57
stress-70 protein, mitochondrial precursor (observed pI 5.6; theoretical pI 5.9)	0.59	0.42
succinate dehydrogenase [ubiquinone] flavoprotein subunit	0.59	0.3
triosephosphate isomerase (pI observed 6.7; theoretical pI 6.5)	0.70	0.64
thioredoxin domain-containing protein 12	0.64	0.7
RhoA precursor	0.65	0.6

***MDAMB231 cells***		

3-hydroxyisobutyrate dehydrogenase, mitochondrial	0.64	0.73

aldose reductase	1.64	1.40

alpha enolase	0.28	absent

annexin A1 (observed pI 6.6; theoretical pI 6.6)	1.67	1.42

annexin A1 (observed pI 6.4; theoretical pI 6.6)	1.24	3.08

annexin A4	1.52	1.63

cathepsin D precursor	0.55	0.52

cell division cycle protein 42	1.76	2.04

chloride intracellular channel protein 1 (observed pI 5.1; theoretical pI 5.1)	1.58	1.98

chloride intracellular channel protein 1 (observed pI 5.3; theoretical pI 5.1)	1.61	1.97

Cleavage stimulation factor 64 kDa subunit	0.28	0.35

cofilin 1/2	0.62	0.71

complement component 1 Q subcomponent-binding protein, mitochondrial	0.41	0.63

Cytochrome b-c1 complex subunit 1, mitochondrial	0.71	0.63

Cytochrome c-type heme lyase	0.35	0.37

dihydrolipoamide S-acetyltransferase, component of PDH complex	0.60	0.68

DJ-1	0.34	0.44

elongation factor 1-delta	0.85	0.24

endoplasmic reticulum protein ERp29 precursor	1.36	1.60

eukaryotic translation initiation factor 3 subunit I	0.70	0.73

Ezrin	2.31	1.77

GDP dissociation inhibitor 2	1.6	1.54

gelsolin precursor (identified in 3 spots as fragment)	1.77	1.80

glutathione S-transferase Pi	1.56	1.84

glutathione S-transferase omega-1	1.65	1.57

glycyl-tRNA synthetase	1.91	1.41

GrpE protein homolog 1, mitochondrial precursor	0.67	0.76

heat shock protein 27	1.33	1.57

heterogeneous nuclear ribonucleoprotein F	0.72	0.95

heterogeneous nuclear ribonucleoprotein H1	1.91	2.32

heterogeneous nuclear ribonucleoprotein K	0.41	0.24

heterogeneous nuclear ribonucleoproteins C1/C2	0.56	0.91

high mobility group protein B1	2.59	1.94

interferon-induced GTP-binding protein Mx2	1.24	1.59

Ku70 antigen	0.46	0.35

lactoyl-glutathione lyase	1.82	2.02

lamin-A/C	0.36	0.45

LIM and SH3 domain protein 1	1.73	1.75

macrophage-capping protein (identified in two spots)	2.00	1.98

minichromosome maintenance protein 7	0.44	0.28

moesin	0.53	1.02

MutS homolog 2	0.35	0.16

nucleophosmin	0.61	0.22

peroxiredoxin 2	1.56	1.58

peroxiredoxin 3	1.63	1.81

plexin-D1 precursor	0.30	0.53

pre-mRNA-processing factor 19	0.76	0.48

prohibitin	1.75	1.59

proliferating cell nuclear antigen	0.15	0.49

proteasome activator complex subunit 1	1.20	1.40

proteasome activator complex subunit 3	0.49	0.39

protein NDRG1	1.69	1.58

putative ATP-dependent Clp protease proteolytic subunit, mitochondrial	0.59	0.76

Ran-specific GTPase-activating protein	1.54	1.66

Ras GTPase-activating protein-binding protein 1	0.54	0.48

reticulocalbin-1 precursor (identified in two spots as fragment)	0.47	0.64

reticulocalbin-1 precursor	0.58	0.78

stomatin-like protein 2	0.64	0.52

stress-70 protein, mitochondrial (observed pI 5.9; theoretical pI 5.9)	0.61	0.74

stress-70 protein, mitochondrial precursor (observed pI 5.4; theoretical pI 5.9)	0.59	0.42

synaptic vesicle membrane protein VAT-1 homolog	5.14	4.55

RhoA precursor	0.49	0.65

TRAP1	1.54	1.76

triosephosphate isomerase (pI observed 6.2; theoretical pI 6.5)	2.37	2.14

triosephosphate isomerase (pI observed 6.5; theoretical pI 6.5)	0.77	0.59

tropomyosin 1 alpha chain isoform 4	0.31	0.44

vinculin	2.12	2.69

zyxin	2.39	0.82

The cell treatment occurred with either 8 μg/mL lovastatin lactone or hydroxy acid for 48 hours. The factor change is presented below with values above 1 representing an increase and values below 1 representing a decrease in protein expression as compared with controls. In some cases (annexin 1, chloride intracellular channel protein 1, stress-70 protein, triose phosphate isomerase) more then one spot was assigned to one protein. This happens when proteins undergo a post-translational modification as indicated by a shift in the spot's isoelectric point (pI). In these cases, an observed and a theoretical pI values were provided.

### Small GTPases mediated signal transduction

Small GTPase family members, some of which are known to modulate Ras protein signal transduction, have been described in the literature as major targets of statins other than HMG-CoA reductase [27,28].

**Figure 3:**
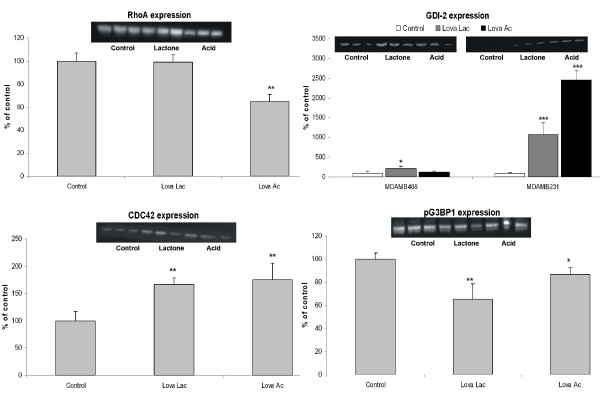
**Western blot analysis of proteins involved in small GTPase-mediated cell signaling**. Breast cancer cell lines MDAMB231 and MDAMB468 were treated with 8 μg/mL lovastatin lactone (Lova Lac) or lovastatin acid (Lova Ac) for 48 hours. For key proteins, western blot analysis was performed based on MDAMB231 cell extracts (for Ras homolog gene family member A (RhoA), cell division cycle 42 (CDC42) and GTPase-activating protein SH3-domain-binding protein 1 - phospho form (pG3BP1)), otherwise both cell lines are shown. Densitometry data were normalized based on the amount of β-actin. Data are presented as means ± standard deviations (n = 3) *P < 0.05;**P < 0.05; ***P < 0.001). Gel images were cropped to improve the clarity and conciseness of the presentation. GDI-2, Rho GDP dissociation inhibitor 2.

Our proteomics data revealed a decrease in total expression of RhoA (Table 1). In addition to the total expression, a western blot analysis on membrane-bound, geranylgeranylated RhoA in MDAMB231 cells was performed and it was found that lovastatin acid caused a significant decrease in the expression of this activated RhoA form, and that only a slight decrease was caused by the lactone (Figure 3). Our data also showed that the expression of GDP dissociation inhibitor 2 (GDI-2), a protein stabilizing the inactive RhoA form, experienced a significant increase and was more pronounced in MDAMB231 than in MDAMB468 cells (Figure 3). Lovastatin also induced downregulation of unmodified and G3BP1 (Table 1, Figures 2a and 3; down-regulation of phospho-G3BP1 only with lovastatin lactone) and cofilin 1/2 proteins (Figure 2a), and an overexpression of CDC42 protein (Figure 3).

### Inhibition of cell proliferation and cell-cycle activity

**Figure 4:**
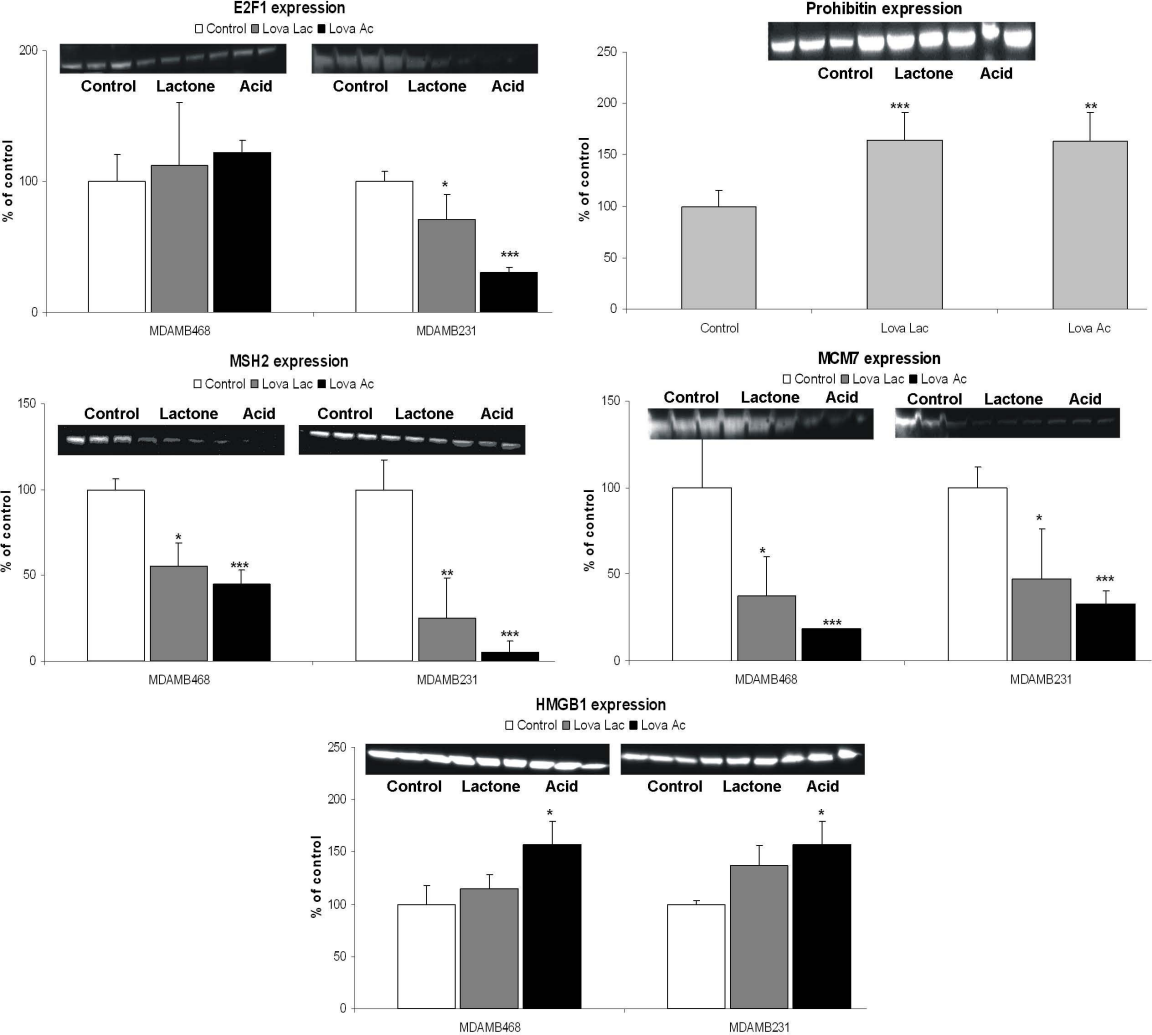
**Western blot analysis of proteins involved in regulation of the cell cycle including the modulation of the E2F1-Rb activity**. Breast cancer cell lines MDAMB231 and MDAMB468 were treated with 8 μg/mL lovastatin lactone (Lova Lac) or lovastatin acid (Lova Ac) for 48 hours. Western blot analysis of prohibitin was performed based on MDAMB231 cell extracts, otherwise both cell lines are shown. Densitometry data were normalized based on the amount of β-actin. Data are presented as means ± standard deviations (n = 3) *P < 0.05;**P < 0.05; ***P < 0.001. Gel images were cropped to improve the clarity and conciseness of the presentation. HMGB1, high-mobility group box 1; MCM7, minichromosome maintenance protein 7; MSH2, MutS homolog 2.

**Figure 5:**
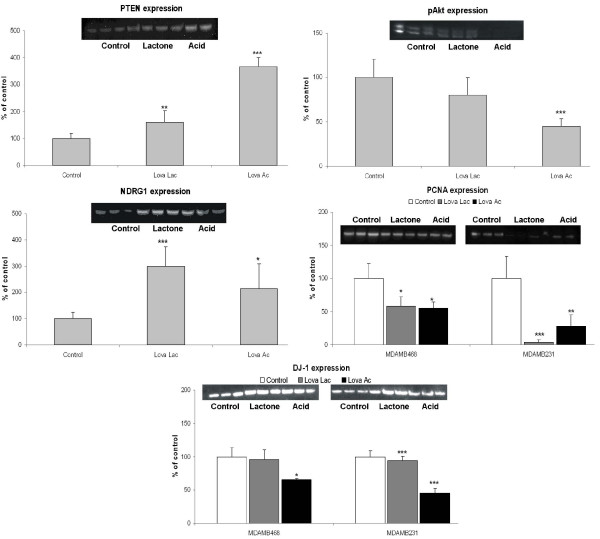
**Western blot analysis of proteins involved in regulation of apoptosis and AKT-signaling**. Breast cancer cell lines MDAMB231 and MDAMB468 were treated with 8 μg/mL lovastatin lactone (Lova Lac) or lovastatin acid (Lova Ac) for 48 hours. For key proteins, western blot analysis of phosphatase and tensin homolog (PTEN), pAkt and N-myc downstream regulated gene 1 (NDRG1) was performed based on MDAMB231 cell extracts, otherwise both cell lines are shown. Densitometry data were normalized based on the amount of β-actin. Data are presented as means ± standard deviations (n = 3) *P < 0.05;**P < 0.05; ***P < 0.001). Gel images were cropped to improve the clarity and conciseness of the presentation. PCNA, proliferating cell nuclear antigen.

Several proteins present in breast cancer cells that are involved in regulation of cell proliferation and cell-cycle activity were significantly altered when exposed to lovastatin. Changes in the expression of the two E2F activity related cell-cycle regulatory proteins prohibitin and MCM7, were also detected. Although the expression of prohibitin increased nearly two-fold (Table 1 and Figure 4), the expression of MCM7, an essential component of the replication helicase complex [29], decreased to 28% of control (Table 1 and Figure 3). Lovastatin-induced DNA damage also had an impact on damage repair regulating pathways. We observed a downregulation of a representative member of DNA-mismatch repair (MMR) systems, MSH2 (Figure 4 and Table 1). Expression of PCNA is downregulated by both forms of lovastatin in MDAMB231 cells, with a stronger reduction in presence of the lactone form (Table 1 and Figure 5).

### Cell death

In both cell lines, lovastatin treatment was accompanied by the loss of cell viability. Functional clustering facilitated the identification and subsequent inclusion of a large group of proteins related to the apoptosis signaling. These included: TNF type 1 receptor-associated protein (TRAP-1), 70 kDa subunit of Ku antigen (Ku70), disulfide isomerase ER-60, DJ-1 (PARK-7; Figure 5), cofilin 1/2, heat shock 27 kDa, HMGB1, glutathione S-transferase Pi, annexins A1 and A4, and nucleophosmin (Table 1).

### Cellular metabolism

Lovastatin treatment altered the expression of proteins involved in the regulation of metabolic processes such as pentose-phosphate pathway (NADP metabolic process): 3-hydroxyisobutyrate dehydrogenase, 6-phosphogluconolactonase, triosephosphate isomerase 1; glycolysis: triosephosphate isomerase 1, alpha enolase, dihydrolipoamide S-acetyltransferase; and tricarboxylic acid cycle activity as indicated by decreased expression of succinate dehydrogenase (ubiquinone) flavoprotein subunit (SDHA) and dihydrolipoamide S-acetyltransferase. ATP citrate lyase, an enzyme involved in synthesis of acetyl-CoA, was downregulated as well (Table 1 and Figure 2b).

### Lovastatin induced oxidative stress

The expression of ROS scavengers peroxiredoxin 2 and peroxiredoxin 3 was upregulated, while the expression of a protein related to the family of thioredoxins, the thioredoxin domain-containing protein 12, was down-regulated (Table 1). An increase in expression levels of two isoforms of glutathione S-transferase, GST-Pi and GST omega-1, was observed (Table 1). Both of these isoforms are active in the detoxification of ROS-induced damage (Figure 2b).

### Correlation of proteomic data with western blot protein expression analysis

In order to confirm the two-dimensional gel electrophoresis proteomics mass spectrometry data, western blot analysis was performed on selected proteins, the results of which are presented in Figures 3 to 5. When not performed on both cell lines, the analysis was performed only on the more sensitive of the two, the MDAMB231. The results obtained from the western blot analysis corresponded well with the results from the proteomics database search. The expression of the small GTPases, GDI-2 and CDC42, showed an increase in MDAMB231 cells. Analysis of the expression of the membrane-bound, active RhoA surprisingly indicated no change after exposure to lovastatin lactone, in contrast to a significant decrease during treatment with lovastatin acid. In the protein group associated with the E2F1 pathway, the expression of E2F1, as well as MSH2, MCM7 and HMGB1 was more pronounced in the lovastatin acid group than in the lovastatin lactone treatment group. Time-dependent changes were, again, more prominent in MDAMB231 than in MDAMB468 cells. The same specific trend towards higher sensitivity of MDAMB231 cells to lovastatin acid continued in the expression of proteins related to Akt signaling. Although the expression of PTEN increased, its associated regulator protein DJ-1 was down-regulated, as was pAkt itself. Conversely, NDRG1, an Akt downstream target, was upregulated by lovastatin lactone and acid.

### Metabonomic analysis

#### Energy producing pathways: glycolysis and Krebs cycle

**Figure 6:**
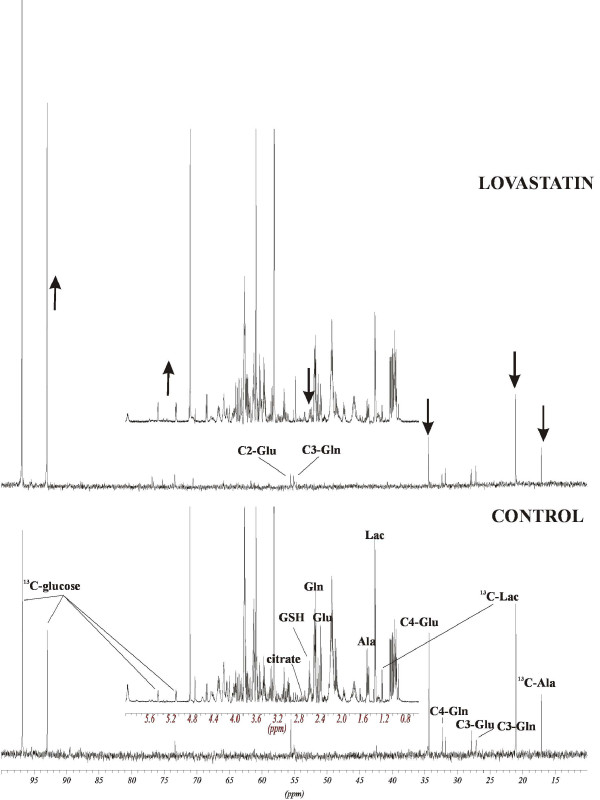
**Changes in intracellular ^13^C-labeled -alanine, -lactate, -glucose and -glutamine signals in MDAMB468 cells treated with 8 μg/mL lovastatin acid for 48 hours**. ^13^C-NMR spectra with embedded, corresponding ^1^H-NMR spectra are shown (including citrate at 2.52 + 2.69 ppm). Arrows indicate the direction of signal changes (increase or decrease). Ala, alanine; Gln, glutamine; Glu, glutamine; GSH, total glutathione; Lac, lactate.

As revealed by ^1^H-NMR, 48 hour incubation of MDAMB468 cells with 8 μg/mL lovastatin lactone or lovastatin acid strongly inhibited glycolytic activity by decreasing the *de novo *production of ^13^C-alanine and ^13^C-lactate. The ^13^C-lactate concentrations (mean ± standard deviation) decreased to 41 ± 8% of control (*P *< 0.001, n = 3) during lovastatin lactone exposure and 56 ± 3% of control (*P *< 0.005, n = 3) during lovastatin acid exposure (Table 2 and Figure 6). Lovastatin lactone and acid also induced a strong reduction in the Krebs cycle activity, as measured through the ^13^C-enrichment of Krebs cycle products, such as glutamine and glutamate. Concentration of C4-glutamate decreased from 474 ± 72 nmol/g in controls to 91 ± 11 nmol/g in lovastatin lactone (*P *< 0.001, n = 3) and to 111 ± 17 nmol/g (*P *< 0.001, n = 3) in lovastatin acid-treated cells (Table 2 and Figure 6). Furthermore, lovastatin acid reduced the concentration of citrate, a direct Krebs cycle intermediate to 30 ± 11% of control (*P *< 0.005, n = 3, Table 2 and Figure 6). The reduction in the activity of these two major glucose metabolizing processes was accompanied by an accumulation of intracellular glucose (Table 2 and Figure 6). In regards to surrogate markers for ROS formation, ^1^H-NMR analysis of cell extracts revealed a highly significant decline in total cellular glutathione concentrations (from 2595 ± 168 nmol/g in controls to 871 ± 72 and 1149 ± 78 nmol/g in lovastatin lactone and acid treated cells; *P *< 0.001, n = 3, Table 2 and Figure 6), suggesting an increase in oxidative damage.

**Table 2:** Intracellular concentrations (nmol/g cell weight) of ^13 ^C-labeled endogenous metabolites (glycolysis and TCA cycle intermediates, glucose) and lipid metabolites (choline-containing phospholipids, cholesterol)

	Control	Lovastatin lactone	Lovastatin acid
**^13^C-lactate**	612 ± 36	252 ± 51**	343 ± 15**
**glycolysis int**	845 ± 21	369 ± 13***	467 ± 52***
**TCA cycle int**	913 ± 232	189 ± 28**	213 ± 56**
**glucose_intracell_**	1918 ± 382	2691 ± 283*	2758 ± 231*
**citrate**	323 ± 46	98 ± 33***	153 ± 35**
**glutathione**	2595 ± 168	871 ± 72***	1149 ± 78**
**choline-PL**	3757 ± 534	2158 ± 275**	2672 ± 542*
***chol C18+C19***	3914 ± 582	2125 ± 289**	2467 ± 351**

The values were calculated based on MDAMB468 cell extracts as assessed by ^1^H-NMR and ^13^C-NMR. The cells were incubated with 8 μg/mL lovastatin lactone or hydroxy acid for 48 hours. Values are presented as means ± standard deviation of three independent experiments. Significance levels: * *P *< 0.05; ** *P *< 0.005; *** *P *< 0.001 were determined by analysis of variance (with *post-hoc *pairwise multiple comparison Tukey-test). chol, cholesterol; choline-PL, choline-containing phospholipids; glu, glutamate; glycolysis int, glycolysis intermediates: ^13^C-lactate+^13^C-alanine, TCA int: TCA cycle intermediates: (C2 + C3 + C4)-glutamate + (C2 + C3)-glutamine.

#### Lipid metabolism

**Figure 7:**
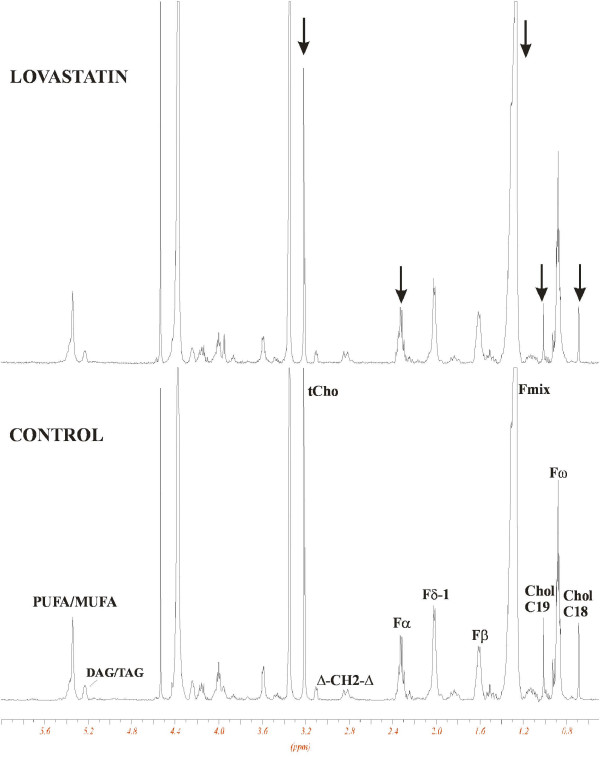
**Representative ^1^H-NMR spectra of MDAMB468 lipid extracts**. Cells were treated with 8 μg/mL lovastatin acid for 48 hours. Arrows indicate the direction of signal changes (decrease). Chol, cholesterol (C18 and C19, CH_3_); Δ (δ), double bond; F, fatty acid side chain; F_α_, F_β_, protons in the fatty acid chain; Fmix: -(CH_2_)n-, tCho, total choline-containing phospholipids.

Both lovastatin forms led to similar changes in the lipid constitution of the cell, causing a reduction in the signals for cholesterol, choline-containing phospholipids and fatty acids (Figure 7). However, the changes were more pronounced in lovastatin lactone-treated cells where the concentration of total choline-containing phospholipids decreased to 57 ± 7% (*P *< 0.005, n = 3), cholesterol C18 to 55 ± 6% (*P *< 0.005, n = 3), cholesterol C19 to 54 ± 9% (*P *< 0.05, n = 3), and concentrations of different unsaturated fatty acids declined to 50 to 65% of control values (Table 2 and Figure 7).

## Discussion

Although the beneficial effects of HMG-CoA reductase inhibitors in lowering cholesterol are well established, their importance in the area of cancer therapeutics is only now beginning to gain greater recognition [1,2,10,12]. Normal cells respond to statin inhibition of HMG-CoA reductase activity through a feedback upregulation of sterol- and lipid-synthesizing gene programs, including the low-density lipid receptor [30]. Cancer cells usually exhibit elevated levels of HMG-CoA reductase and low-density lipid receptor. Thus, cancer cells are potentially more sensitive than normal cells to the isoprenoid-depleting effects of statins [31]. In this study, we used a combination of 2DE-proteomic and NMR-based metabonomic strategies to further investigate the molecular mechanisms by which lovastatin exhibits its reported antitumor activity.

**Figure 8:**
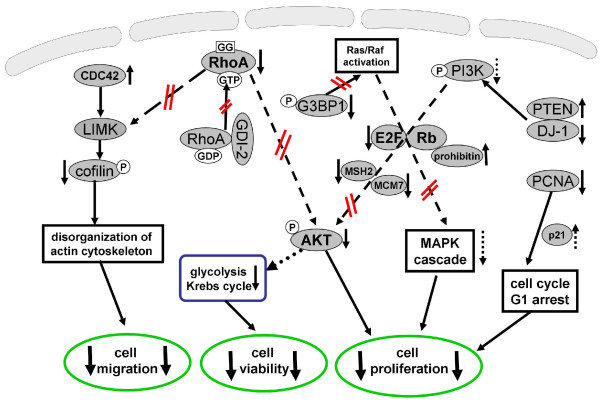
**Schematic diagram summarizing the effects of lovastatin lactone and acid on signaling pathways as found in the present study**. The solid arrows mark the directional change of proteins (up- or down-regulation). Doted arrows mark hypothesized change in protein expression/activity. CDC42, cell division cycle 42; G3BP1, GTPase-activating protein SH3-domain-binding protein 1; GDI-2, Rho GDP dissociation inhibitor 2; LIMK, LIM domain kinase; MAPK, mitogen-activated protein kinase; MCM7, minichromosome maintenance protein 7; MSH2, MutS homolog 2; NDRG1, N-myc downstream regulated gene 1; p21, cyclin-dependent kinase inhibitor 1A; PCNA, proliferating cell nuclear antigen; PI3K, phosphoinositide 3-kinase; PTEN, phosphatase and tensin homolog; Rb, retinoblastoma protein; RhoA, Ras homolog gene family member A.

Two estrogen receptor-negative breast cancer cell lines, MDAMB231 and MDAMB468, were treated for 48 hours with 8 μg/mL lovastatin lactone or lovastatin hydroxy acid. Although MDAMB231 cells express PTEN and Rb, MDAMB468 does not express either of these. In regards to their sensitivity to lovastatin, both cell lines exhibited similar IC_50 _values. However, in regard to changes detected by 2DE, MDAMB231 cells demonstrated alterations in a larger number of proteins and presumably a greater sensitivity to lovastatin. After exposure to lovastatin acid or lactone, the majority of proteins detected did not show differences in changes between the two treatment groups. This may partly be supported by previous data, which shows that in a cell culture medium, 80% of the lactone prodrug converts to the acid form within 9 hours and achieves complete conversion within 24 hours [32]. Western blot analysis further confirmed that the observed lovastatin-induced changes in protein expression were more pronounced in the MDAMB231 than the MDAMB468 cells. This suggests that their phenotypic differences (e.g. PTEN, Rb expression) may be responsible for the stronger response to lovastatin. In MDAMB231 cells, the differences between the lovastatin lactone and lovastatin acid were more distinct, in general with lovastatin acid exhibiting greater effects, especially on the GTPase, E2F and AKT signaling pathways (Figures 3, 4, 5 and Figure 8).

Inhibition of the mevalonate pathway by lovastatin suppresses the synthesis of two substrates that provide the isoprenoid moieties for post-translational modifications of diverse proteins: farnesyl and geranylgeranyl diphosphates [33]. This suppresses the essential post-translational processing of proteins regulating cell proliferation and viability [34]. Examples are the Ras and Rho proteins, which require attachment of FPP or GGPP groups prior to their activation and delocalization to the plasma membrane [35]. Several groups have reported that the addition of mevalonate pathway intermediates such as mevalonate, GGPP and partially FPP, can diminish the pro-apoptotic effects of statins [36]. Also, the addition of mevalonate (at 100-fold the lovastatin concentration) has been shown to release the cells from the G1 cell cycle arrest induced by lovastatin and allow for entry into late G1, S and G2/M phases [37]. This points to the predominant role of protein geranylgeranylation in statin-induced apoptosis in cancer cells [10,38,39]. In our study, the addition of mevalonate and GGPP reversed the effects of lovastatin on the inhibition of breast cancer cell proliferation, whereas FPP could only partially rescue cells from the antiproliferative effect of lovastatin. Although FPP lies upstream of GGPP in the mevalonate pathway, the addition of FPP would not be capable of restoring protein geranylgeranylation because a second molecule, isopentenyl pyrophosphate (PPi), is required for the conversion of FPP to GGPP. Isopentenyl PPi is also depleted by statin exposure, and is therefore unavailable to the statin-treated cells.

Small GTPase-proteins are frequently discussed targets of statins [27,28]. Our proteomics data identified RhoA, a protein implicated in the control of cell growth, apoptosis [40] and tumorigenesis [41]. We demonstrated that the translocalization of RhoA in MDAMB231 cells to the membrane was suppressed by lovastatin (Figures 3 and 8). We also observed an increased expression of GDI-2, which stabilizes the non-activated form of RhoA and prevents its relocalization to the membrane and subsequent activation by GGPP (Figure 8). In addition, lovastatin acid treatment changed the expression of Ras-GTPase activating binding protein G3BP1 (down-regulation of its unmodified and its active phospho form) and CDC42 (upregulated; Figures 3 and 8). The latter acts as a signal transduction convergence point in intracellular signaling networks mediating multiple signaling pathways, including tyrosine kinase receptors, heterodimeric G-protein coupled receptors and cytokine receptors [42]. G3BP1 directly associates with the SH3 domain of GTPase-activating protein, functioning as an effector of Ras [43]. Moreover, we identified a decrease of cofilin 1/2, a CDC42 and LIM kinase target protein [44] (Figure 8). Post-translational modification analysis (using the ProteinPilot and special factors: phosphorylation emphasis, paragon search method) revealed that the cofilin form decreased by lovastatin was phosphorylated at S3, S8 and T16. This reduction of the phosphorylated cofilin is in accordance with previous reports [45].

Regulation of the cell cycle including the modulation of Rb-E2F1 activity is the second major signaling pathway affected by lovastatin treatment in breast cancer cells (Figure 8). PCNA, a cell proliferation marker and a control point for DNA repair [46], was found to be significantly down-regulated by lovastatin in both cell lines. Its downregulation has been proven to correlate with the overexpression of p21 and is followed by a G1 arrest in cells [47]. The latter has been shown to occur in cells treated with statins [12,37,48,49], making them popular as agents for reversible synchronization of cells in the G1 phase of the cell cycle [37].

The upregulation of the cell-cycle regulatory protein prohibitin, a tumor suppressor protein able to co-localize with Rb and suppress the E2F1 and p53 transcriptional activity [50], is another novel finding of our study. Despite the observation that prohibitin is upregulated in both cell lines following lovastatin treatment (to a higher degree in MDAMB231 cells), an expected downregulation of E2F1 only occurred in Rb-positive MDAMB231 cells. Therefore, while acting synergistically with Rb in the suppression of E2F1, prohibitin does not seem to impair E2F1 expression alone. As for the downstream targets in the E2F-mediated pathway, we identified changes in both MCM7 [51] and MSH2 [52]. Although MCM7 belongs to the cell cycle DNA checkpoints, MSH2 is a representative member of MMR systems. The expression of both of these was significantly suppressed by lovastatin. Interestingly, the suppression occurred in both cell lines, suggesting that it may not be mediated exclusively through E2F1 reduction, and that perhaps other regulatory pathways are also affected by lovastatin.

Statin-treated breast cancer cells die through apoptosis [12,48,49]. It was therefore not surprising that a large number of identified proteins was associated with the programmed cell death pathway. In addition to prohibitin, RhoB and cofilin 1/2, there was also suppression of TRAP-1 and Ku70 expression. Both of these proteins protect the cells from apoptosis and oxidative stress [53,54]. These data comply with previous reports suggesting that increased oxidative stress may be a cause of statin-induced cytotoxicity in breast cancer [13,49]. Recently, it has been shown that fluvastatin and simvastatin enhance NO levels and increase iNOS RNA and protein expression in breast cancer MCF-7 cells, indicating that iNOS-mediated NO is responsible, in part, for the proapoptotic, tumoricidal, and antiproliferative effect of statins [14]. Furthermore, the cell death of MCF-7 cells incubated with N-acetyl-L-cysteine plus statins could almost be reversed [49], supporting our results that oxidative stress plays an important role in the cell death induced by statins.

In terms of metabolic changes, the downregulation of glycolytical enzymes triosephosphate isomerase, alpha-enolase and dihydrolipoamide acetyltransferase and tricarboxylic acid cycle enzymes such as SDHA represent potential pathways by which lovastatin may induce cell death through the suppression of energy-producing pathways. Glycolysis is the primary energy-producing pathway in cancer cells and is therefore a highly valuable target in anti-cancer therapy [55]. The changes in enzyme expressions correlate with the NMR-based metabolic profiles: decreased production of *de novo *^13^C-lactate, ^13^C-alanine and C4-glutamate and accumulation of intracellular glucose (Figures 6 and 8).

Due to its close relation to anaerobic glycolysis [56], we chose to investigate the role of the protein kinase Akt. A downregulation of the active p-Akt form was detected in both cell lines. One possible mechanism of Akt deactivation involves its regulation by PTEN, inhibiting the ability of phosphatidylinositol 3-kinase to phosphorylate Akt [57]. As expected, we observed an induction of PTEN expression by lovastatin in the PTEN-expressing MDAMB231 cell line (Figures 5 and 8). The induction was more pronounced when the cells were treated with the lovastatin acid than with its lactone form. PTEN itself is known for tumor suppression and frequently mutates in a wide variety of cancers and is functionally involved in their metastatic advancement [58]. The ability of statins to stimulate the overexpression of PTEN and their importance for therapeutic and preventative uses in cancer, diabetes mellitus and cardiovascular disease has been recognized in the past [59-61]. To date, several mechanisms have been discussed including the transcriptional activation of peroxisome proliferator-activated receptor and upregulation of the sterol response element-binding protein [59-61]. In our proteomics data we have identified a protein affected by lovastatin described in the literature as a negative regulator of PTEN [62,63]. This protein, known as DJ-1/PARK7, is an oncogene that cooperates with H-Ras and transforms cells by increasing cell proliferation and resistance to cell cycle arrest [64]. In breast cancer, overexpression of DJ-1 positively correlates with phosphorylated Akt and poor disease prognosis [62]. In both of our breast cancer cell lines (PTEN expressing MDAMB231 and PTEN lacking MDAMB468), lovastatin acid successfully decreased the expression of DJ-1 (Figures 5 and 8). Conversely, lovastatin lactone, previously shown to induce PTEN in a less effective manner than the acid form, failed to decrease DJ-1 expression. This result confirms that the expression of DJ-1 is correlated with the expression of PTEN and suggests that DJ-1 is able to regulate the activity of the Akt kinase even in the absence of PTEN. DJ-1 and PTEN synergistically lowered the expression of the active pAkt form, but only when cells were treated with lovastatin acid. Our results suggest that DJ-1, and not PTEN, might be the key regulator of pAkt expression in lovastatin-treated breast cancer cells. This hypothesis will require further evaluation. The influence of lovastatin is also detected downstream of the DJ-1/PTEN-regulated Akt pathway on the expression of yet another clinically important protein, NDRG1. NDRG1 not only plays an important role in metastatic tumor progression, it has also been observed to slow the advancement of breast cancer in a clinical study and, interestingly, to be regulated by PTEN through an Akt-dependant pathway [65]. The downregulation of NDRG1 occurred in cells treated with either lovastatin lactone or lovastatin acid, indicating that its expression might be regulated through pathways other than the inhibition of pAkt.

### Correlation between metabonomic and proteomic data

Dihydrolipoamide S-acetyltransferase and ATP citrate lyase are enzymes that are involved in the production of acetyl-CoA. A reduction in their expression decreases production of acetyl-CoA. This has a negative effect on fatty acid and cholesterol synthesis. Our NMR data revealed a significant reduction of choline-containing phospholipids, fatty acids and cholesterol concentrations as a result of lovastatin treatment. Additionally, we identified a transporter, the sterol carrier protein-X/2, which is not only involved in cholesterol, fatty acids and phospholipids trafficking [66], but also has a high affinity for isoprenyl pyrophosphates (GGPP, FPP, GPP) [67]. Its downregulation suggests that both, the production of isoprenylated intermediates and their transport are influenced by lovastatin.

## Conclusions

Overall, our data indicated that in the studied breast cancer cells lovastatin lactone and acid affect small GTPase, E2F and AKT signaling pathway (Figure 8). Lovastatin-treated breast cancer cells showed changes in the activity of various small GTPases, primarily through the inhibition of the isoprenylation of RhoA. This inhibition is partially mediated by the stabilization of the non-active RhoA form, which is achieved through an increase in expression of Rho inhibitor GDI-2. Lovastatin decreased the activity of G3BP1, a GTPase that is over-expressed in a number of human malignancies. It can be speculated that this may constitute a novel target for the sensitization of cancer cells to genotoxic stress. Lovastatin also modulated the E2F1 pathway by regulating the expression of prohibitin and Rb and resulted in changes of the E2F-downstream targets MCM7 and MSH2. The deactivation of the AKT-pathway through an upregulation of PTEN and down-regulation of DJ-1 represents an additional target by which lovastatin possibly regulates tumor cell survival and progression. It is important to mention the induction of oxidative stress, suppression of glycolytic and Krebs cycle activity as well as lipid biosynthesis as metabolic consequences to lovastatin exposure.

## Abbreviations

ANOVA: analysis of variance; CDC42: cell division cycle 42; DAVID: Database for Annotation, Visualization, and Integrated Discovery; DTT: dithiothreitol; ER: estrogen receptor; FPP: farnesyl pyrophosphate; G3BP1: phospho-GTPase activating protein binding protein 1; GDI-2: GDP dissociation inhibitor 2; GGPP: geranylgeranyl diphosphate; HMG-CoA: 3-hydroxy-3-methylglutaryl coenzyme reductase; HMGB1: high-mobility group protein B1; iNOS: nitric oxide synthase; Ku70: 70 kDa subunit of Ku antigen; LC: liquid chromatography; MCM7: minichromosome maintenance protein 7; MMR: DNA-mismatch repair; MS: mass spectrometry; MSH2: MutS homolog 2; NDRG1: N-myc downstream regulated gene 1; NMR: nuclear magnetic resonance; NO: nitric oxide; PCA: perchloric acid; PCNA: proliferating cell nuclear antigen; PTEN: phosphatase and tensin homolog; Rb: retinoblastoma; RhoA: Ras homolog gene family member A; ROS: reactive oxygen species; SDHA: succinate dehydrogenase (ubiquinone) flavoprotein subunit; TRAP: TNF type 1 receptor-associated protein; TSP: (Trimethylsilyl) propionic-2,2,3,3-acid.

## Competing interests

The authors declare that they have no competing interests.

## Authors' contributions

JeK carried out the proteomic and metabonomic studies and drafted the manuscript; TS helped with the NMR analyses and cell culture work; VM carried out the cell culture work; UC participated in the study design, coordination, data interpretation and helped to draft the manuscript; JoK helped with proteomics data analysis and statistics, participated in the study design and coordination and helped to draft the manuscript. All authors read and approved the final manuscript.
